# Assessment of Zr Metal–Organic Frameworks (UiO-66) for the Adsorption of *p*‑arsanilic Acid from Natural and Drinking Water

**DOI:** 10.1021/acsomega.5c08933

**Published:** 2025-11-14

**Authors:** Luan F. Passos, Ana Carina S. Conto, Beatris L. Mello, Vicente P. Matos, Juliana S. F. Pereira, Eder C. Lima, Christian W. Lopes, Diogo P. Moraes

**Affiliations:** † 28124 Institute of Chemistry, Universidade Federal do Rio Grande do Sul, Porto Alegre 91501-970, Brazil; ‡ Department of Chemistry, 28122 Universidade Federal do Paraná, Curitiba 81531-980, Brazil

## Abstract

The organoarsenical compounds, such as *p*-arsanilic acid (*p*-ASA) and roxarsone (ROX), are commonly used as veterinary drugs to control intestinal parasites in poultry and swine farms. Because of this, environmental contamination by organoarsenical compounds can occur, primarily affecting water sources, which has raised widespread concern. Metal–organic frameworks (MOFs) are emerging as promising materials for water purification because of their varied crystal structures, extensive surface areas, adjustable pore sizes, and excellent chemical stability. In this study, a zirconium-based MOF (UiO-66) was synthesized, and its structural properties were analyzed using XRD, SEM, TGA, NMR, and BET surface area measurements. The performance of Zr-MOF was evaluated for removing the organoarsenical compound (*p*-ASA) from natural and drinking water. Batch adsorption experiments were carried out to assess the effects of pH, adsorption time, analyte concentration, and potential interferences. The adsorption efficiency did not significantly change across the pH range of 4–8. The isotherm studies showed that the adsorption mechanism was best described by the Langmuir model (*R* ^2^ 0.9880), with a maximum adsorption capacity of 542.6 mg g^–1^. This indicates the presence of uniform adsorption sites and the formation of a monolayer. The adsorption kinetics studies revealed that the Avrami model fitted the data well (*R* ^2^ 0.9971), suggesting a complex adsorption mechanism primarily based on physisorption (*k*_AV_ 1.33). The proposed method demonstrates that UiO-66 is highly effective at removing *p*-ASA from natural and drinking water, even in the presence of coexisting ions, providing a sustainable approach to remediating contaminated water sources.

## Graphical abstract

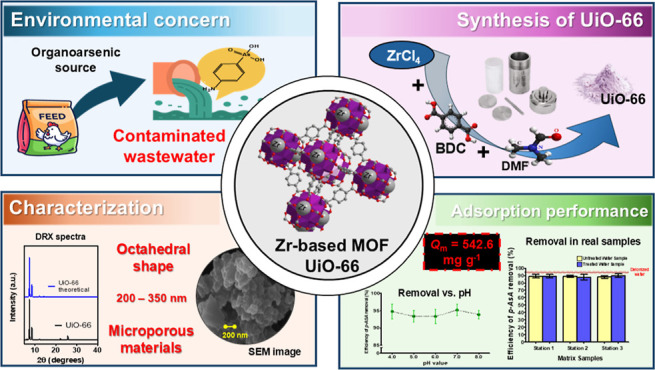

## 1 Introduction

Inorganic arsenic is classified as a harmful chemical element, mainly to humans and the environment.^1^ However, arsenic can be found in various chemical compounds and oxidation states. The literature indicates that trivalent inorganic arsenic in the aqueous phase exhibits the highest toxicity. Furthermore, organoarsenic compounds, particularly water-soluble ones, may possess carcinogenic potential. These compounds can degrade under environmental conditions, turning into highly toxic inorganic substances arsenic. Aromatic organoarsenic compounds, such as *p*-arsanilic acid (*p*-ASA) or roxarsone (ROX), are typically added to poultry and swine feed to control coccidial intestinal parasites and enhance feed efficiency.^2,3^ Moreover, these compounds are not readily absorbed through the gastrointestinal tract, and since no chemical structural changes have been observed, they can be present in the excreta.^4^ As a result, arsenic levels in animal waste can increase, leading to environmental contamination of natural water sources in areas of intensive factory farming.^5,6^ Therefore, it is crucial to develop methods that can effectively remove organoarsenic compounds from natural and drinking water.

Multiple studies have examined the removal of organoarsenic compounds from water with adsorbents.^7,8^ Metal–organic frameworks (MOFs) are an innovative class of versatile materials composed of metal clusters and adaptable organic ligands. They possess various crystal structures, customizable pore sizes, and extensive surface areas. Usually, MOFs exhibit high thermal stability and chemical resistance, remaining stable across a broad pH spectrum.^9–12^

Recently, MOFs have been studied for removing arsenic or organoarsenic compounds from water.^13^ The metal sites within MOF clusters play a key role in interacting with arsenical species. These sites form strong Lewis acid–base interactions with oxyanions by binding arsenic through coordinate covalent bonds.^14^ The classification of MOFs mainly depends on the type of metal ion, such as zirconium.

Zirconium-based (Zr) MOFs are highly promising for arsenic removal because of their exceptional stability, high adsorption capacity, and strong affinity for arsenic oxyanions. This strong interaction is especially clear in UiO-66 MOF, which show increased arsenic retention. This is achieved by forming direct ZrO–As coordinate bonds, with hydroxyl groups and benzenedicarboxylate ligands playing important roles in the binding process.^14^

Yang et al. reported the design and synthesis of a Zr/Eu bimetallic metal–organic framework (MOF) containing missing-linker defects, subsequently functionalized with amine groups, for the detection and adsorption of trace amounts of *p*-ASA (limit of detection: 0.72 mg L^–1^). The enhanced adsorption affinity was attributed to the material’s high surface area and the abundance of defect sites. Furthermore, the Eu-doped MOF demonstrated applicability not only in the adsorption but also in the luminescence-based detection of *p*-ASA. The adsorption efficiency remained above 94% after the fifth regeneration cycle. However, this evaluation was conducted using a standard solution of 2 mg L^–1^ *p*-ASA in the absence of matrix components or competing ions.^15^ Similar results were reported by Xu et al., who demonstrated that structural defects generated additional Zr–OH sites in the metal nodes, which are essential for the formation of a bidentate binuclear configuration. In addition, amine groups were shown to establish hydrogen bonds with both *p*-ASA and roxarsone (ROX), acting as secondary interactions that further stabilized the As–O–Zr complex.^16^

Lin et al. investigated a series of Zr-based MOFs (DUT-67, UiO-66, UiO-67, MOF-808, MOF-808F, NU-1000, and NU-1000B) for the adsorptive removal of *p*-ASA and ROX from aqueous solutions. Among these, MOF-808 exhibited the highest adsorption capacity. However, it demonstrated lower adsorption specificity and reusability compared to MOF-808F. Notably, the most significant results were obtained with MOFs whose structures are not analogous to that of UiO-66, which is the focus of the proposed work.^17^

Typically, MOF synthesis is performed in batch mode. However, Xiao et al. developed a high-throughput continuous microfluidic strategy to produce the Zr-based MOF UiO-66 with a hierarchical porous structure, enabling efficient and rapid removal of *p*-ASA. Compared with conventional hydrothermal methods, microfluidic synthesis offers a simpler, more scalable, and highly effective approach for *p*-ASA removal. The microfluidic MOF exhibits an adsorption capacity of up to 333.3 mg g^–1^, and when combined with its potential for industrial-scale production, it represents a major advancement for environmental applications and water purification.^18^

The excellent biocompatibility of MOFs is a major advantage for their use in drug delivery and gas therapy for anticancer treatments. In this context, Wang et al. reported the development of a nanoplatform based on the incorporation of manganese carbonyl (MnCO) into a Zr-based MOF, enabling the delivery of CO as a therapeutic gaseous molecule for cancer therapy. In vitro and in vivo studies demonstrated that this multifunctional nanoplatform not only inhibited tumor growth through a synergistic effect, but also introduced a novel strategy for integrating starvation, gas, and chemodynamic therapies within a single material.^19^

The proposed study aimed to synthesize and characterize Zr-metal–organic frameworks and to evaluate their performance as adsorbents for removing organoarsenical compounds (*p*-ASA) from natural and drinking water.

## 2 Experimental Section

### 2.1 Solutions and Samples

All solutions were prepared using purified water (Milli-Q Integral System, Elix Advantage Water Purification System, Merck Millipore, USA), which exhibited a minimum resistivity of 18.2 MΩ.cm at 25 °C. For this study, a 500 mg L^–1^ stock solution of *p*-ASA (Merck, Germany) was prepared by dissolving the corresponding salt in purified water. Subsequently, the solutions were stored in conditions that prevented light exposure and maintained controlled temperatures.

The pH solutions were carefully adjusted to a range of 4–8 using minimal volumes of 0.1 mol L^–1^ HCl or 0.1 mol L^–1^ NaOH. Solutions containing coexisting ions were prepared using single standards of Na (Vetec, Brazil), Mg (SpecSol, Brazil), and PO_4_^3–^ (SPEX CertiPrep, USA). The fluoride (F^–^) stock solution was prepared by dissolving NaF salt (Merck, Germany). A stock solution of dissolved organic carbon (DOC) was prepared by dissolving citric acid, oxalic acid, and humic acid (Merck, Germany) at a concentration of 500 mg L^–1^.

Water samples were collected from the inlet (untreated) and outlet (treated) points of three water treatment stations in Porto Alegre, Brazil. Prefiltration was performed to remove suspended particulate matter utilizing 0.45 μm Nylon syringe filters (Merck, Millipore).

All laboratory materials, including polypropylene vials, glassware, and other flasks, were cleaned prior to use by soaking in a 10% (v/v) nitric acid (HNO_3_) solution for a specified period, followed by thorough rinsing with ultrapure water.

### 2.2 Instrumental

The adsorption process was carried out using a mechanical agitator (model Fisatom 713, Brazil). For isotherm and kinetic studies, a temperature-controlled horizontal shaker (OXY 303T, OXYLAB, Brazil) was utilized. After the batch adsorption, the remaining adsorbate was separated from the solid phase by centrifugation using a Kasvi K14 5000 M centrifuge. The residual *p*-ASA concentration was subsequently measured using a PG Instruments T90+ UV/vis Spectrophotometer (PG Instruments, United Kingdom).

The pH of the solutions was measured using a Metrohm (Switzerland) 781 pH/Ion Meter. An H^+^ ion-selective electrode (Metrohm, Switzerland) and an Ag/AgCl reference electrode with a Pt1000 temperature sensor (Metrohm, Switzerland) were components of the instrument setup.

The dissolved carbon content was measured using a carbon analyzer (Multi N/C 2100S, Analytik Jena, Germany) with an autosampler (AS60, Analytik Jena, Germany). The analysis was performed by combusting samples at 850 °C to produce CO_2_, which was then detected using an infrared detector with a Focus Radiation System (NDIR, Analytik Jena, Germany) for accurate measurement. Solid materials were weighed with an analytical balance (XS105, Mettler Toledo, USA), capable of measuring up to 120 g with a resolution of 0.00001 g.

### 2.3 Synthesis and Characterization of UiO-66

The synthesis of UiO-66 followed the methodology of Santiago-Portillo et al.^20^ with adaptations. Briefly, 0.1675 g of terephthalic acid (BDC), 0.244 g of zirconium chloride, and 3 mL of dimethylformamide were dissolved, and the solution was transferred to a stainless steel autoclave lined with PTFE. The autoclave was then maintained in an oven at 120 °C for 24 h. Afterward, the product was vacuum filtered, washed with DMF, and dried in an oven at 60 °C overnight. The resulting MOF was named UiO-66.

The synthesized MOF was characterized using an Ultima IV (Rigaku, Japan) X-ray diffractometer with Cu K radiation (λ 1.54056) and a step size of 0.05°. Nitrogen physisorption analyses were performed using a Micromeritics TriStar II 3020 instrument. The solids were pretreated at 120 °C overnight before analysis. The obtained isotherms were processed using the BET method to determine the specific surface area, the t-plot method to obtain the micropore volume, and the Gurvich method to determine the total pore volume. The elemental analysis (CHN) was carried out using an elemental analyzer (2400 Series II CHNS/O, PerkinElmer, USA) with a thermal conductivity detector. Thermogravimetric analysis was conducted on a TA Instruments Q50 device under a compressed air atmosphere, heating from room temperature to 900 °C at a gas flow rate of 40 mL min^–1^. Nuclear magnetic resonance spectroscopy (NMR) of ^13^C was performed on an Agilent DD2 spectrometer. The ^13^C spectrum of the UiO-66 was acquired at 125.7 MHz resonance frequency using a CP-MAS sequence. The FTIR spectra were obtained using a Cary 630 spectrometer (Agilent) coupled to an attenuated total reflectance (ATR) accessory equipped with a ZnSe crystal.

### 2.4 Adsorption Experiments

The effect of pH was explored using solutions set to various pH levels (4.0, 5.0, 6.0, 7.0, and 8.0). For each pH value, 5 mg of UiO-66 was introduced into 5 mL of a 100 mg L^–1^ *p*-ASA solution. Mechanical agitation was performed at 100 rpm for 90 min across all aqueous suspensions. Afterward, the samples were centrifuged, and the supernatant was analyzed with a UV-vis spectrophotometer at 253 nm.

Adsorption isotherm experiments for *p*-ASA were carried out using concentrations ranging from 5 to 185 mg L^–1^. The solutions were stirred at 120 rpm for 20 h. Subsequently, the adsorbent material was separated by centrifugation at 4000 *g* for 10 min. The concentration of *p*-ASA remaining in the supernatant was subsequently analyzed, with a 1:10 dilution performed if needed.

The kinetics of adsorption were conducted using 100 mg L^–1^ *p*-ASA solutions. The adsorption contact times that were assessed included 1, 2, 3, 4, 5, 10, 15, 30, 45, 60, 75, 90, 105, 120, 150, 180, and 240 min.

Adsorption interference studies were evaluated by the addition of coexisting ions at concentrations of 10 and 100 mg L^–1^. Additionally, the influence of dissolved organic carbon (DOC) was evaluated at a concentration of 10 and 100 mg L^–1^. For real sample analysis, samples were artificially contaminated with *p*-ASA at a concentration of 100 mg L^–1^.

### 2.5 Data Analysis

Adsorption efficiency was determined using eq 1.

1 $$\%,removal=\frac{C_{0}-C_{eq}}{C_{0}}x100\%$$

where *C*_0_ is the initial concentration of *p*-ASA, and *C*_e_ is the *p*-ASA concentration at equilibrium.

Analysis of adsorption isotherms was performed with the Langmuir eq 2 and the Freundlich eq 3 models.

2 $$q_{e}=\frac{Q_{máx}.K_{L}.C_{e}}{1+K_{L}.C_{e}}$$

3 $$q_{e}=K_{F}.C_{e}^{n}$$

In these models, *q*_e_ denotes the equilibrium *p*-ASA adsorption capacity. *Q*_max_ is the maximum equilibrium adsorption capacity, and *k*_L_ and *k*_F_ correspond to the Langmuir and the Freundlich constants, respectively.

The adsorption kinetics were analyzed using the pseudo-first-order, pseudo-second-order, and Avrami fractional-order models (see eqs 4, 5, and 6).

4 $$q_{t}=\left[1-\exp\left(-k.t\right)\right]$$

5 $$q_{t}=\frac{K.q_{e}^{2}.t}{1+q_{e}.k.t}$$

6 $$q_{t}=q_{e}.\left\{1-\exp{\left[-\left(k.t\right)\right]}^{n}\right\}$$

In these models, q_t_ represents the quantity of *p*-ASA adsorbed at a specific time *t*, while *q*_e_ denotes the adsorption capacity at equilibrium. *k* is the kinetic constant for the given order, *t* signifies the adsorption contact time, and *n* refers to the fractional adsorption order.

## 3 Results and Discussion

### 3.1 UiO-66 Characterization

The MOF was characterized by determining its chemical parameters and structural properties. Figure 1 shows the X-ray diffraction results of the UiO-66 synthesized in this work. It can be observed that the reference UiO-66 was successfully synthesized, as its diffractogram matches the theoretical pattern. However, there is a broad peak at ∼6° that has been attributed to the presence of reo-phase nanoregions or missing cluster defects.^21^ SEM images revealed a consistent octahedral morphology with particle sizes ranging from 200 to 350 nm, as shown in Figure 2.

**Figure 1:**
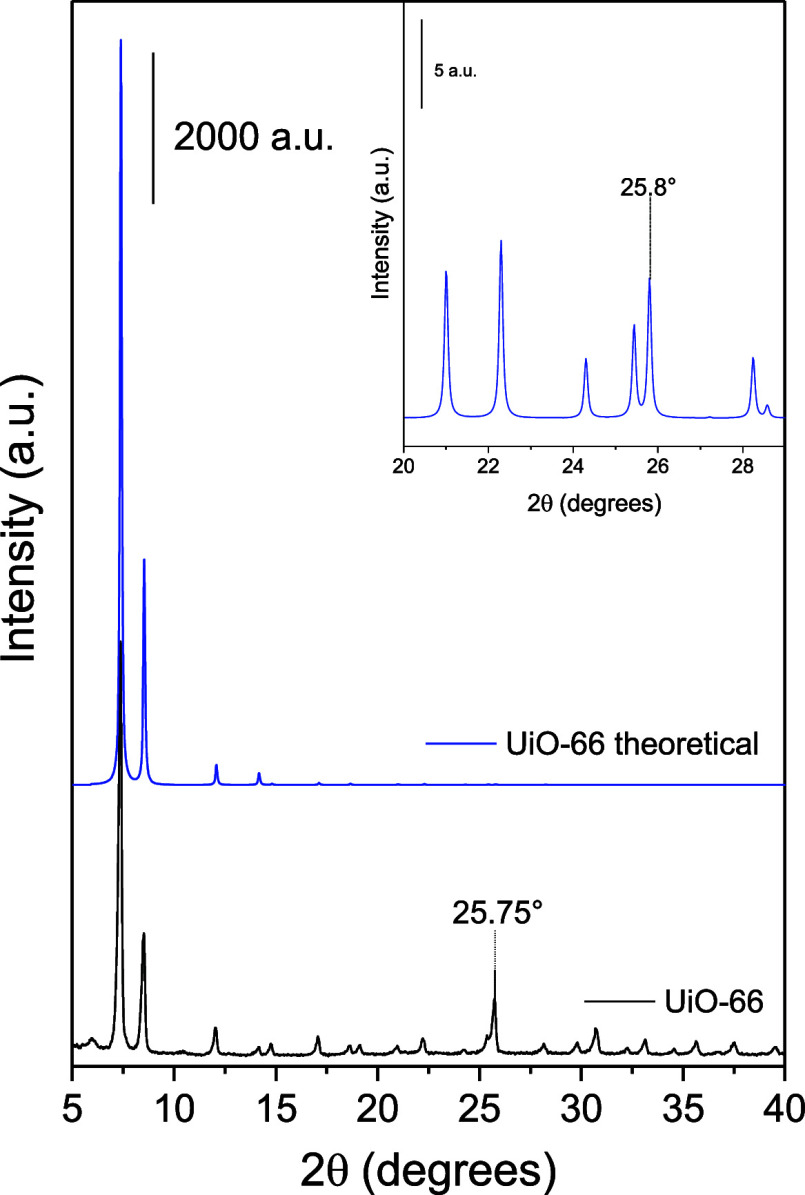
X-ray diffraction patterns of theoretical (blue) and synthesized (black) UiO-66. The inset shows a zoomed-in view of the 20–29° region, where the well-resolved peak at ∼25.8° can be seen.

**Figure 2:**
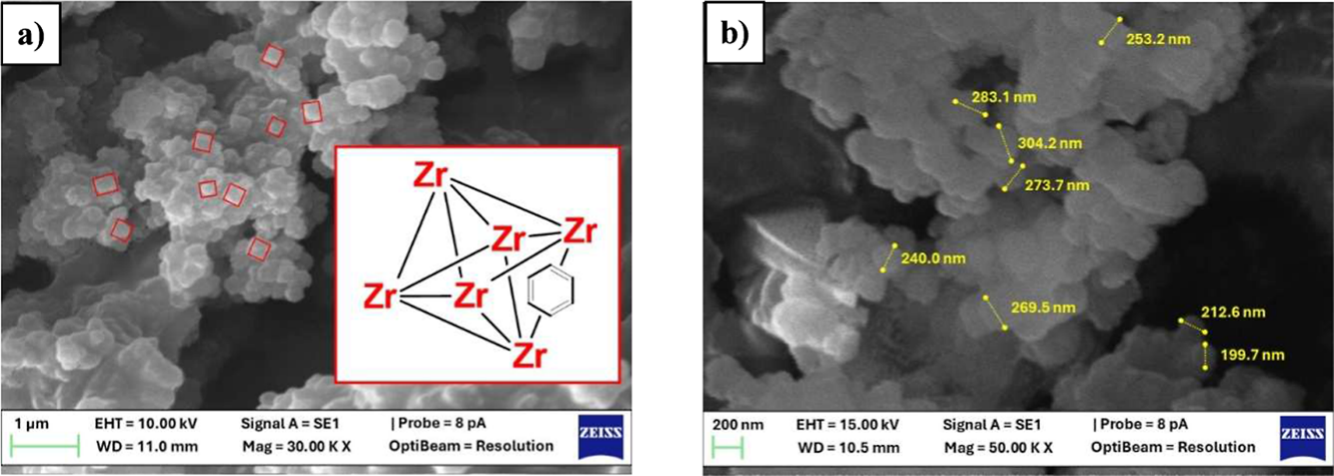
SEM images of UiO-66. Scale bar: (a) 1 μm and (b) 200 nm.

Figure 3 presents the N_2_ adsorption/desorption isotherm of UiO-66 MOF. The material shows a type I isotherm, characteristic of microporous materials.^22^ The specific surface area (SBET), micropore volume (*V*_μ_), and total pore volume (VTP) of the MOF are listed in Table 1. The BET analysis showed a high surface area of 915 m^2^/g, which is advantageous for adsorption applications. The values of the textural properties align with the literature, indicating that the MOF was successfully crystallized.

**Figure 3:**
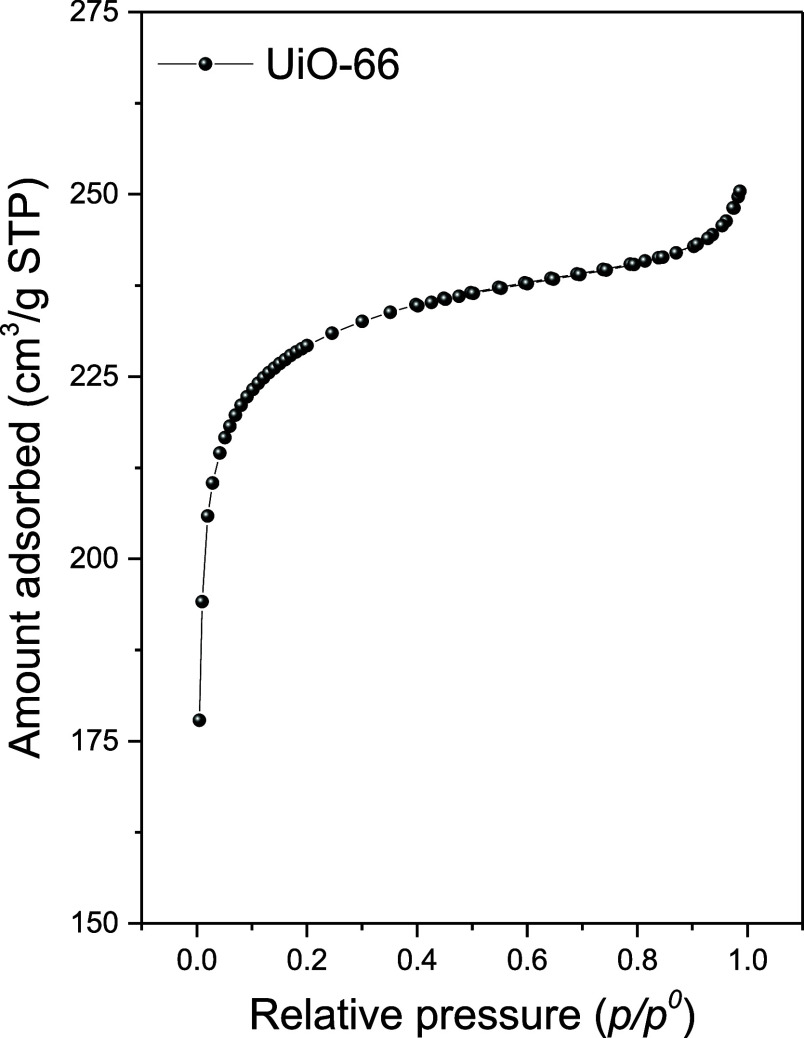
N_2_ physisorption isotherm of UiO-66.

**Table 1:** Chemical Analysis and Textural Properties of the UiO-66 Used in This Work

sample ID	% Zr^a^	% C^b^	% N^b^	*S*_BET_ (m^2^/g)	*V*μ (cm^3^/g)	VTP (cm^3^/g)
UiO-66	25.2	26.4	2.6	914	0.28	0.38

The ^13^C CP-MAS spectrum of UiO-66 (Figure S1) demonstrates the preservation of the terephthalate linker, as indicated by the presence of signals at 135 and 144 ppm, corresponding to the two distinct carbon environments in the aromatic ring of the BDC ligand. Additionally, the carboxylate functional group is responsible for the resonance observed at 177 ppm.^23^ By using the amount of residue (∼34 wt %) obtained from the TG analysis (Figure S2), we were able to determine the amount of ZrO_2_ and consequently the percentage of zirconium in the material, which was 25.2%.^24^ The amount of carbon and zirconium are slightly smaller than the range found in the literature for other UiO-66 materials,^25^ which can be caused by the presence of connectivity defects in our synthesized sample. Although the Zr content alone cannot be considered definitive evidence of structural defects, the measured Zr content of 25.2% in our sample (lower than the theoretical value expected for defect-free UiO-66) correlates with the appearance of an additional XRD reflection at approximately 6°. This feature has previously been attributed to missing-cluster defects (reo-phase nanoregions) by Shearer et al.^21^ As emphasized in the Supporting Information of their study, both missing-linker and missing-cluster defects result in linker deficiencies. The absence of a cluster implies the loss not only of the Zr_6_O_6_^12+^ secondary building unit but also of the 12 BDC^2–^ linkers that would ideally connect it to neighboring clusters. This structural deficiency accounts for the reduced Zr and C contents relative to the ideal UiO-66 composition and provides a plausible explanation for the occurrence of defects in our sample.

### 3.2 Effect of pH

The evaluation of pH is a critical parameter in adsorption experiments because it can influence both the molecular structure of UiO-66 and *p*-ASA. The pH level can affect the surface charge distribution of UiO-66, thereby impacting the adsorption capacity of the MOF. UiO-66 demonstrated desirable adsorptive capacity at lower pH levels due to surface protonation, which results in a positively charged surface that enhances the adsorption of anionic species. Conversely, at higher pH levels, the adsorption efficiency often decreases because hydroxide ions occupy active sites on the MOF surface ions.^26^

Furthermore, the presence of *p*-ASA in aqueous solutions depends on pH, which influences its different forms. *p*-ASA has three p*K*a values (2.00, 4.02, and 8.98) and can exist in four different forms: protonated (HASA^+^), zwitterionic (*p*-ASA), and two deprotonated forms (ASA^–^ and ASA^2–^). The cationic form (HASA^+^) only appears under highly acidic pH conditions. Conversely, the two anionic forms are the main species under basic conditions. In this study, experiments were conducted within a pH range of 4–8 to examine *p*-ASA’s adsorption behavior on UiO-66. Typically, most environmental samples (wastewater, sludge, and soil) have pH values ranging from above 4 to below 8. Under the evaluated conditions, the main form of *p*-ASA is the anionic ASA^–^ form (the arsenate group begins to deprotonate at pH levels greater than 4), with a characteristic peak around 253 nm (Figure S3). According to the results shown in Figure 4, pH has a negligible effect on the adsorption of *p*-ASA by UiO-66, with adsorption efficiencies of 94.1 ± 0.8% across a pH range from 4 to 8. The statistical evaluation was performed using One-Way ANOVA (with 95% confidence), and no significant differences were observed among the studied pH levels.

**Figure 4:**
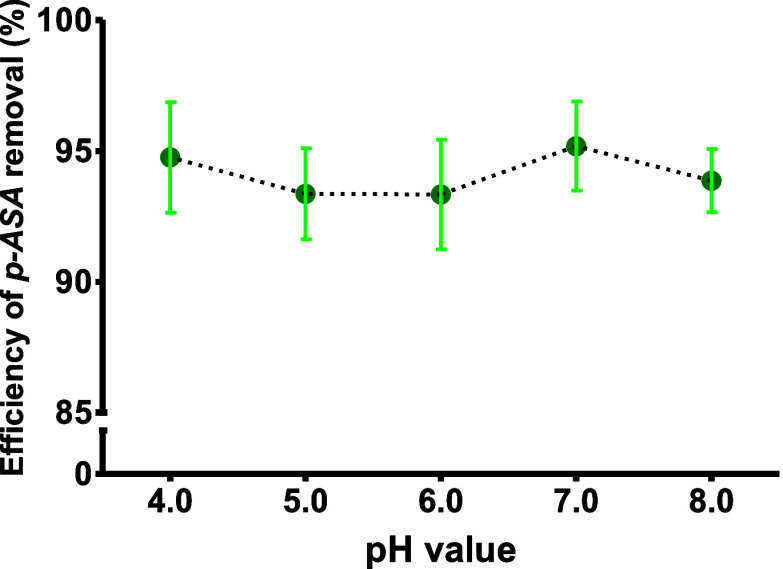
Effect of pH on *p*-ASA adsorption by UiO-66. Error bars represent the standard deviation (*n* = 3). Experimental conditions: 5 mg UiO-66, 5 mL of a 100 mg L^–1^ *p*-ASA, and adsorption time of 90 min.

Moreover, the X-ray diffraction patterns (Figure S4) confirm that the UiO-66 retains its crystalline structure under different pH conditions. The characteristic diffraction peaks of the UiO-66 framework are observed at 2θ ≈ 7.4°, 8.5°, and 25.7°, corresponding to the (111), (200), and (600) crystal planes, respectively. This Comparison of the patterns indicates that the overall crystal structure is preserved at both pH 4 and pH 8, although slight variations in peak intensities suggest minor differences in crystallinity. The peaks observed at 7.4°and 8.5° indicate that the MOF is preserved across the pH range of 4–8, maintaining a symmetric cubic structure with high crystallinity.

### 3.3 Adsorption Isotherms

The evaluation of adsorption behavior was investigated using the models of Langmuir and Freundlich. The adsorption isotherms of *p*-ASA by UiO-66 are shown in Figure 5. All the parameters evaluated in the equilibrium of adsorption are shown in Table 2.

**Figure 5:**
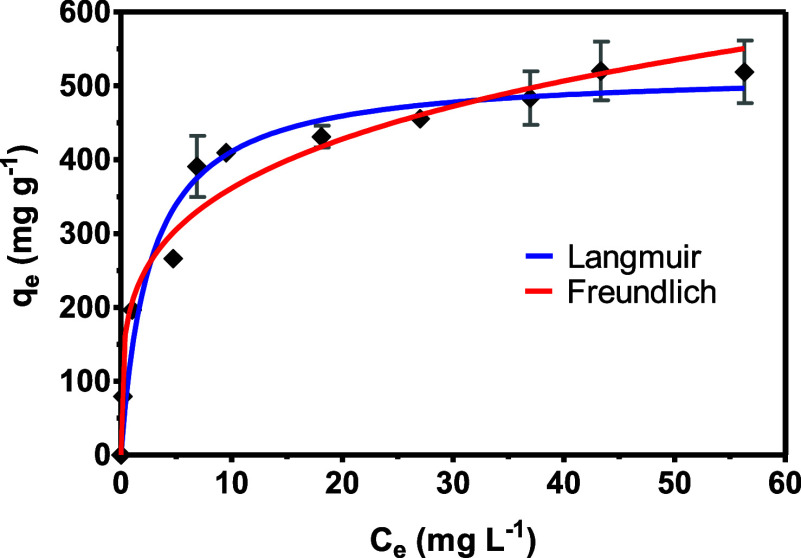
Adsorption isotherm of *p*-ASA fitted to nonlinear Langmuir (blue) and Freundlich (red) models. Experimental conditions: 5 mg of UiO-66, 5 mL of *p*-ASA solution, and adsorption time of 24 h.

**Table 2:** Comparison of Nonlinear Langmuir and Freundlich Model Parameters for the Adsorption of *p*-ASA by UiO-66

parameter	Langmuir model	Freundlich model
*R* ^2^	0.9880	0.9770
*k*_L_ (L mg^–1^)	0.3130	
*k*_F_ (mg g^–1^ (mg L^–1^)^−1/nF^)		247.9
*Q*_m_ (mg g^–1^)	542.6	
*N*		5.160

The UiO-66 showed a high adsorption capacity, and a high coefficient of determination (*R* ^2^) was obtained using both Langmuir and Freundlich isotherm models. However, the Langmuir isotherm model demonstrated a better fit with a higher *R* ^2^ value based on curve fitting criteria. The Langmuir model describes an adsorption process where a single layer of adsorbate forms, all adsorption sites are equivalent, and adsorbate molecules do not interact with each other. Additionally, the constant k_L_ is useful for estimating the affinity of the adsorbent for the analyte in solution; this parameter is especially important when analyzing low-concentration analytes.

Further calculations indicated that the maximum adsorption capacity of UiO-66 for *p*-ASA was 542.6 mg g^–1^, which is lower than the 649 mg g^–1^ reported by Lin et al.^17^ for another Zr-based MOF (MOF-808). Nevertheless, the synthesized UiO-66 exhibited an outstanding adsorption capacity when compared to previously reported studies using UiO-66, which achieved an adsorption capacity of 59.17 mg g^–1^.^16^ This result can be explained by the presence of missing cluster defects in UiO-66, which enhances its adsorption capacity. To confirm the molecular structure of UiO-66 before and after the adsorption process, FTIR analysis was carried out. As shown in Figure S5, the spectrum of UiO-66 exhibits a band at 1350–1450 cm^–1^, corresponding to the symmetric stretching vibration of C–O bounds, and bands in the range of 1480–1600 cm^–1^, which are assigned to the C=C stretching vibrations of aromatic rings. Additionally, the bands observed between 740–680 cm^–1^ are attributed to the Zr–O stretching vibrations characteristic of the UiO-66. After adsorption, new characteristic peaks emerge at 678, 702, and 728 cm^–1^, corresponding to the formation of Zr–O–As bonds. These new features provide clear evidence of a strong chemical interaction between the Zr sites of MOF and *p*-ASA molecules during the adsorption process, confirming successful binding and possible coordination within the MOF.

### 3.4 Adsorption Kinetics of *p*-ASA on UiO-66

The adsorption of *p*-ASA by UiO-66 is influenced by adsorption kinetics; thus, it is carefully investigated in this work to optimize the residency time and study the adsorption rate. The synthesized MOF achieved an equilibrium of adsorption within 10 min (efficiency of adsorption higher than 90%).

Figure 6 illustrates this result, showing that *p*-ASA quickly adsorbs onto the material surface upon contact. As available adsorption sites decrease, the rate of adsorption slows down. Eventually, adsorption saturates and reaches a plateau after 45 min. This behavior is primarily due to the accessible adsorption sites on UiO-66, which initially hold the *p*-ASA molecules effectively. This explanation aligns with the BET and morphological findings. To explore the rate-controlling steps of *p*-ASA ion transfer and the physicochemical interactions involved in adsorption, several kinetic models were applied to analyze the experimental data. Table 3 displays the parameters derived from fitting experimental data with various kinetic models. The high *R* ^2^ value of 0.9971 indicates that the data calculated using the Avrami model closely match the experimental results. Overall, the Avrami equation is deemed the most precise for describing *p*-ASA adsorption, with the constant *k*_AV_ (1.33) representing the adsorption rate. This observation aligns with the earlier hypothesis, which links *p*-ASA adsorption to active sites created by defects like missing linkers on the material surface. Additionally, this model has been effectively used to explain complex kinetic processes involving multiple adsorption mechanisms, as demonstrated by the material studied here. Therefore, *p*-ASA adsorption by UiO-66 involves ion transfer and physicochemical reactions occurring on the outer surface.

**Figure 6:**
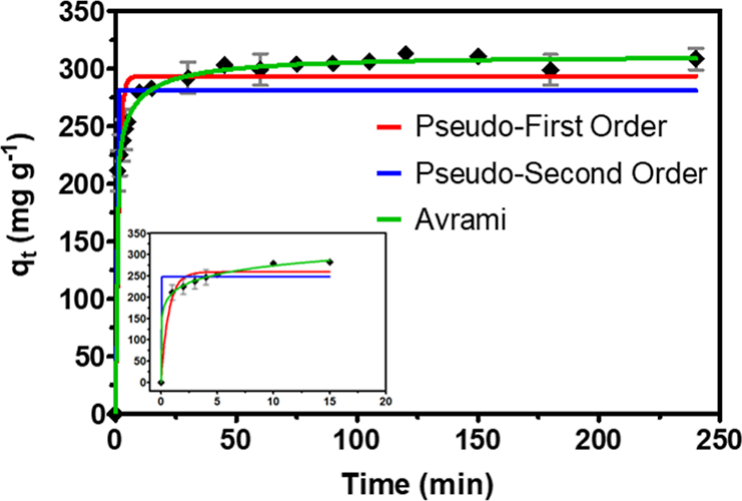
Adsorption kinetics of *p*-AsA fitted to nonlinear models: pseudo-first order (red), pseudo-second order (blue), and Avrami (green). Experimental conditions: 5 mg of UiO-66, 5 mL of a 75 mg L^–1^ of *p*-ASA.

**Table 3:** Fitting Results of *p*-ASA Adsorption Kinetics over UiO-66 Using Pseudo-First Order, Pseudo-second Order, and Avrami

parameter	pseudo-first order	pseudo-second order	Avrami
*R* ^2^	0.9177	0.8106	0.9971
*q*_e_ (mg g^–1^)	293.4	280.9	310.4
*k*_1_ (L mol^–1^)	0.8215		
*k*_2_ (min^–1^)		1.241	
*k*_AV_			1.33

### 3.5 The Evaluation of Coexisting Ions Effect on the Adsorption Process

The presence of coexisting ions usually shows a negative effect on the efficiency of adsorption. Some ions commonly found in natural and drinking water samples include Na^+^, Mg^2+^, PO_4_^3–^, and F^–^. Therefore, the study of the influence of common ions on the *p*-ASA adsorption was carried out. The impact of these ions in concomitance with *p*-ASA was evaluated at concentration levels of 10 and 100 mg L^–1^, as illustrated in Figure 7.

**Figure 7:**
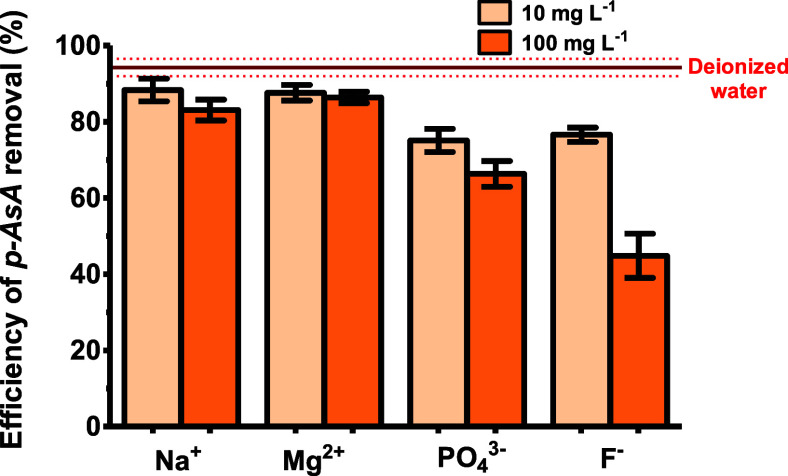
Evaluation of the efficiency of *p*-ASA adsorption by UiO-66 in the presence of coexisting ions. Error bars represent the standard deviation (*n* = 3). Experimental conditions: 5 mg of UiO-66, 5 mL of a 100 mg L^–1^ of *p*-ASA, and a adsorption time of 45 min.

The results show a slight decrease in the adsorption efficiency of *p*-ASA in the presence of cationic ions, regardless of their concentration. These results suggest that the active sites on the material surface are either neutral or positively charged, and the presence of cationic species presents a negligible effect on the adsorption process.

The anionic ions demonstrated a more substantial decrease in the efficiency of *p*-ASA adsorption by UiO-66. Among the anionic ions examined, F^–^ induced a more pronounced decrease in the efficiency of adsorption compared to PO_4_^3–^. The smaller ionic size of the F^–^ ions can facilitate their penetration into the internal pore structure of the adsorbent, thereby reducing the number of available active sites for *p*-ASA adsorption. Furthermore, since UiO-66 has been previously utilized for PO_4_^3–^ adsorption, the observed decrease in the efficiency of adsorption suggests that the uptake of PO_4_^3–^ anions by UiO-66 can be associated with a mechanism of surface complexation based on an ion exchange process.^27^

The presence of dissolved organic carbon (DOC) was evaluated in this study at concentrations of 0, 10, and 100 mg L^–1^ of carbon. Additionally, the effect of the molecular structure of carbon was examined using citric acid, oxalic acid, and humic acid. The impact of dissolved organic carbon content on the efficiency of *p*-ASA adsorption is shown in Figure 8.

**Figure 8:**
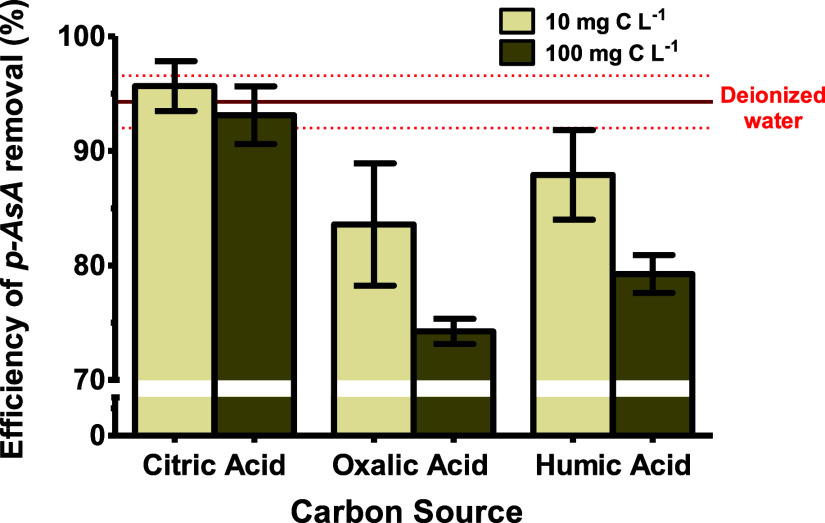
Evaluation of the efficiency of *p*-ASA adsorption by UiO-66 in the presence of dissolved organic carbon (DOC). Error bars represent the standard deviation (*n* = 3). Experimental conditions: 5 mg of UiO-66, 5 mL of a 100 mg L^–1^ of *p*-ASA, and adsorption time of 45 min.

The decrease in adsorption efficiency is due to the different affinities of organic molecules for the active sites. In this study, all organic acids existed in their anionic form at pH levels between 5 and 6. As previously noted, these anionic species greatly affect adsorption efficiency by competing with *p*-ASA for the active sites of UiO-66. Additionally, the stronger effect of oxalic acid can be linked to its smaller molecular size and additional interactions with the MOF linkers, like H-bonding and van der Waals forces. These interactions influence both the occupation of active sites and the presence of missing defects on the MOF surface, leading to a notable reduction in *p*-ASA adsorption.

### 3.6 Performance of UiO-66 for the Removal of *p*-ASA from Real Samples of Natural and Drinking Water

The presence of coexisting ions and microorganisms in natural and drinking water samples is a known source of interference in adsorption-based removal processes. These concomitant species have the capacity to compete with *p*-ASA for active sites on the UiO-66 adsorbent, often resulting in reduced adsorption efficiency.

The *p*-ASA removal efficiency was assessed on water samples from three different water treatment stations using UiO-66 as an adsorbent. For each station, the adsorption process was evaluated using both untreated (inlet of water treatment plant) and pretreated water samples (outlet of water treatment plant). As shown in Figure 9, the results indicate that the purification process has no substantial effect on the efficiency of the adsorbent material to remove *p*-ASA.

**Figure 9:**
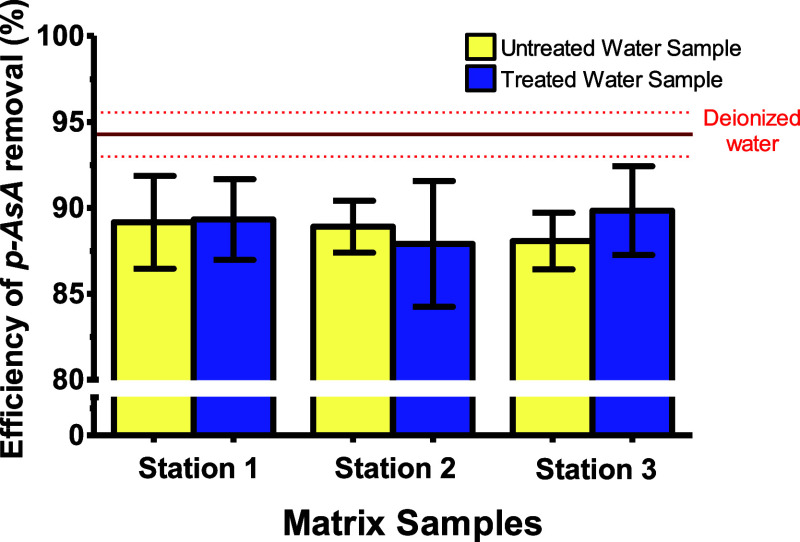
Application of the *p*-AsA adsorption process in real water samples (natural and drinking) using UiO-66. Error bars represent the standard deviation (*n* = 3). Experimental conditions: 5 mg of UiO-66, 5 mL of real samples artificially contaminated with 100 mg L^–1^ of *p*-ASA, and adsorption time of 45 min.

The removal efficiency of *p*-ASA in deionized water (matrix-free) was 94.4%. A statistically significant decrease in removal efficiency was observed in all real water samples when compared to the matrix-free sample. Despite this reduction, the efficiencies remained highly satisfactory. This demonstrates the adsorbent’s robust performance even in the presence of competitive species, such as inorganic ions and dissolved organic matter, which are known to compete for available adsorption sites. Quantitative measurements of *p*-ASA by molecular absorption using UV–vis spectrometry (Figure S6) were compared with the total arsenic determination by graphite furnace atomic absorption spectrometry (GF AAS) to evaluate the accuracy of the method. No statistically significant difference was observed between the total arsenic concentration determined by UV–vis spectrometry (through conversion of *p*-ASA concentration into total arsenic concentration) and GF AAS (*t*-test; *p* < 0.05), confirming the reliability and precision of both analytical techniques. These results indicate that UV–vis spectrometry provides an accurate and practical approach for quantifying *p*-ASA in the samples, consistent with the results obtained by the more sensitive GF AAS method.

Moreover, the recyclability was evaluated after the adsorption process using real samples of natural and drinking water spiked with 25 mg L^–1^ of *p*-ASA. The adsorption efficiency did not change significantly after four consecutive cycles, demonstrating the high adsorption capacity and stability of UiO-66. However, the desorption procedures using 0.01 mol L^–1^ HCl or 0.01 mol L^–1^ NaOH did not exceed 50%, even when the process was extended overnight. This behavior may be attributed to the strong binding interactions between the adsorbent and the adsorbate. The effect of HCl as eluent was more pronounced compared to NaOH. It is important to note that the interferences observed in the UV–vis spectra provide sufficient evidence that the MOF structure collapses when exposed to 0.01 mol L^–1^ NaOH (pH 12). However, after the fifth cycle, the adsorption efficiency decreased to approximately 60%. This result indicates that the UiO-66 structure may lose some linkers during repeated use. It is important to note that this evaluation was conducted with real samples containing additional chemical species, whereas most studies in the literature report results obtained using *p*-ASA standard solutions in the absence of a matrix.

## 4 Conclusions

In this work, a UiO-66 metal–organic framework (MOF) was successfully synthesized and characterized. The material exhibited high porosity, large crystal size, and high specific surface area. Its adsorption capacity was evaluated for the removal of *p*-ASA from natural and drinking water. The results of the isothermal adsorption experiments demonstrated that the Langmuir isotherm model was the most suitable to describe the adsorption behavior of *p*-ASA on UiO-66. Our experimental results found that the removal capacity of *p*-ASA by UiO-66 was higher than previously reported in the literature (*Q*_m_ 542.6 mg g^–1^). Moreover, the kinetic adsorption study was best fitted by the Avrami model, which suggests a complex mechanism of adsorption based on a multistep approach.

The interference by the presence of coexisting ions was evaluated, and a negligible effect was observed for cationic ions. However, the effect of anionic species, such as fluoride and phosphate, decreases the adsorption capacity of UiO-66, suggesting an anion-exchange mechanism on the surface of the material. The effect of dissolved organic carbon was most pronounced upon the addition of oxalic acid, resulting in a 30% reduction in the adsorption efficiency.

Moreover, from an application standpoint, using real water samples, the results were remarkable compared to the adsorption procedure conducted in purified water. Considering that arsenic contamination is a global concern, this work provides a reliable, effective, and straightforward method for the removal of *p*-ASA from natural and drinking water.

## Supplementary Material



## Notes

^13^C CP-MAS NMR spectrum of the UiO-66 (Figure S1). Thermogravimetric curve of the UiO-66 (Figure S2). UV–vis spectra of *p*-ASA at pH = 7.0 (Figure S3). X-ray diffraction patterns of the synthesized UiO-66, UiO-66 at pH 4, and UiO-66 at pH 8 (Figure S4). Comparison of FTIR spectra of UiO-66 before and after the adsorption of *p*-ASA (Figure S5). Calibration plot to quantitative measurements of *p*-ASA using UV–vis spectrometry (Figure S6) (PDF)

## References

[1] Agency for Toxic Substances and Disease Registry. Public health statement Toxicological Profile for Arsenic; U.S. Department of Health and Human Services: Atlanta, 2007; pp 1–14.

[2] Liu X., Zhang W., Hu Y., Cheng H. (2013). Extraction and Detection
of Organoarsenic Feed Additives and Common Arsenic Species in Environmental
Matrices by HPLC–ICP-MS. Microchem. J..

[3] Iwuozor K. O., Akpomie K. G., Conradie J., Adegoke K. A., Oyedotun K. O., Ighalo J. O., Amaku J. F., Olisah C., Adeola A. O. (2022). Aqueous
Phase Adsorption of Aromatic Organoarsenic Compounds: A Review. J. Water Process Eng..

[4] Gupta S. K., Le X. C., Kachanosky G., Zuidhof M. J., Siddique T. (2018). Transfer of
Arsenic from Poultry Feed to Poultry Litter: A Mass Balance Study. Sci. Total Environ..

[5] Czaplicka M., Jaworek K., Klyta J. (2019). Determination
of Selected Organoarsenic
Compounds by SPME/GC-MS in Aquatic Samples. Desalin. Water Treat..

[6] Liu X., Zhang W., Hu Y., Hu E., Xie X., Wang L., Cheng H. (2015). Arsenic Pollution of
Agricultural
Soils by Concentrated Animal Feeding Operations (CAFOs). Chemosphere.

[7] Kumar P., Bansal V., Kim K.-H., Kwon E. E. (2018). Metal-Organic Frameworks
(MOFs) as Futuristic Options for Wastewater Treatment. J. Ind. Eng. Chem..

[8] Ahmadijokani F., Ghaffarkhah A., Molavi H., Dutta S., Lu Y., Wuttke S., Kamkar M., Rojas O. J., Arjmand M. (2024). COF and MOF
Hybrids: Advanced Materials for Wastewater Treatment. Adv. Funct. Mater..

[9] Kirlikovali K. O., Hanna S. L., Son F. A., Farha O. K. (2023). Back to the Basics:
Developing Advanced Metal–Organic Frameworks Using Fundamental
Chemistry Concepts. ACS Nanosci. Au.

[10] An Y., Lv X., Jiang W., Wang L., Shi Y., Hang X., Pang H. (2024). The Stability
of MOFs in Aqueous Solutions-Research Progress and
Prospects. Green Chem. Eng..

[11] Dhaka S., Kumar R., Deep A., Kurade M. B., Ji S.-W., Jeon B.-H. (2019). Metal–Organic
Frameworks (MOFs) for the Removal
of Emerging Contaminants from Aquatic Environments. Coord. Chem. Rev..

[12] Beydaghdari M., Saboor F. H., Babapoor A., Asgari M. (2022). Recent Progress in
Adsorptive Removal of Water Pollutants by Metal-Organic Frameworks. ChemNanoMat.

[13] Alam E. (2025). Metal–Organic
Frameworks (MOFs) for Arsenic Remediation: A Brief Overview of Recent
Progress. RSC Adv..

[14] Wang C., Liu X., Chen J. P., Li K. (2015). Superior Removal of Arsenic from
Water with Zirconium Metal-Organic Framework UiO-66. Sci. Rep..

[15] Yang T., Nath I., Mohamed W. A., Deng M., Cai Z., Wu Z., Van Der
Voort P. (2025). Highly Efficient Detection and Removal
of Low-Concentration p-Arsanilic Acid in Water Using a New Defect-Engineered
UiO­(Zr, Eu)-66-(NH_2_)_2_. Chem. Eng. J..

[16] Xu Y., Lv J., Song Y., Zhou X., Tian C., Hong X., Cai Y., Zhao C., Lin Z. (2019). Efficient Removal of Low-Concentration
Organoarsenic by Zr-Based Metal-Organic Frameworks: Cooperation of
Defects and Hydrogen Bonds. Environ. Sci.-Nano.

[17] Lin Z.-J., Zheng H.-Q., Zeng Y.-N., Wang Y.-L., Chen J., Cao G.-J., Gu J.-F., Chen B. (2019). Effective and Selective
Adsorption of Organoarsenic Acids from Water over a Zr-Based Metal-Organic
Framework. Chem. Eng. J..

[18] Xiao G., Zhang J., Zheng M., Chen L., Afewerki S., Dai M., Guo J. (2025). High-Throughput Continuous Microfluidic Magnifiable
Manufactured Microstructured MOFs-Mediated Micropollutants Mitigation
(M7). Sep. Purif. Technol..

[19] Wang Y., Liang X., An J., Pu J., Meng Y., Bai Y., Yu W., Gao Y., Chen T., Yao Y. (2024). H_2_O_2_-Triggered CO Release Based on Porphyrinic Covalent
Organic Polymers for Photodynamic/Gas Synergistic Therapy. Chem. Commun..

[20] Santiago-Portillo A., Navalón S., Álvaro M., García H. (2018). Generating
and Optimizing the Catalytic Activity in UiO-66 for Aerobic Oxidation
of Alkenes by Post-Synthetic Exchange Ti Atoms Combined with Ligand
Substitution. J. Catal..

[21] Shearer G. C., Chavan S., Bordiga S., Svelle S., Olsbye U., Lillerud K. P. (2016). Defect Engineering: Tuning the Porosity
and Composition
of the Metal–Organic Framework UiO-66 via Modulated Synthesis. Chem. Mater..

[22] Thommes M., Kaneko K., Neimark A. V., Olivier J. P., Rodriguez-Reinoso F., Rouquerol J., Sing K. S. W. (2015). Physisorption
of Gases, with Special
Reference to the Evaluation of Surface Area and Pore Size Distribution
(IUPAC Technical Report). Pure Appl. Chem..

[23] Bennett T. D., Todorova T. K., Baxter E. F., Reid D. G., Gervais C., Bueken B., Van De Voorde B., De Vos D., Keen D. A., Mellot-Draznieks C. (2016). Connecting
Defects and Amorphization in UiO-66 and
MIL-140 Metal–Organic Frameworks: A Combined Experimental and
Computational Study. Phys. Chem. Chem. Phys..

[24] Valenzano L., Civalleri B., Chavan S., Bordiga S., Nilsen M. H., Jakobsen S., Lillerud K. P., Lamberti C. (2011). Disclosing the Complex
Structure of UiO-66 Metal Organic Framework: A Synergic Combination
of Experiment and Theory. Chem. Mater..

[25] Katz M. J., Brown Z. J., Colón Y. J., Siu P. W., Scheidt K. A., Snurr R. Q., Hupp J. T., Farha O. K. (2013). A Facile Synthesis
of UiO-66, UiO-67 and Their Derivatives. Chem.
Commun..

[26] de
Oliveira A., Leite H. G., Parreiras V. A., Silva J. C. d. M. (2024). Arsenic Adsorption in Subunits of Metal-Organic Frameworks—A
DFT Approach to Assist Water Treatment. ChemistrySelect.

[27] Gu Y., Xie D., Ma Y., Qin W., Zhang H., Wang G., Zhang Y., Zhao H. (2017). Size Modulation
of
Zirconium-Based Metal Organic Frameworks for Highly Efficient Phosphate
Remediation. ACS Appl. Mater. Interfaces.

