# Supplementary material for: Assessment of Zr Metal–Organic Frameworks (UiO-66) for the Adsorption of p‑arsanilic Acid from Natural and Drinking Water
Source: ACS Omega. 2025 Nov 14;10(46):56578–86. doi: 10.1021/acsomega.5c08933 (PMC12658837; doi:10.1021/acsomega.5c08933)
Supplement: Supplementary file 1 [file ao5c08933_si_001.pdf]

Assessment of Zr metal-organic frameworks (UiO-66) for the adsorption of *p*-arsanilic acid from natural and drinking water

Luan F. Passos[1], Ana Carina S. Conto[1], Beatris L. Mello[1], Vicente P. Matos[1], Juliana S. F. Pereira[1], Eder C. Lima[1], Christian W. Lopes[2], Diogo P. Moraes[1*]

[1]*Institute of Chemistry, Universidade Federal do Rio Grande do Sul, 91501-970, Porto Alegre, RS, Brazil*

[2]*Department of Chemistry, Universidade Federal do Paraná, 81531-980, Curitiba, PR, Brazil*

*Corresponding author. Phone: +55 51 3308 7203

E-mail address: diogo.moraes@ufrgs.br

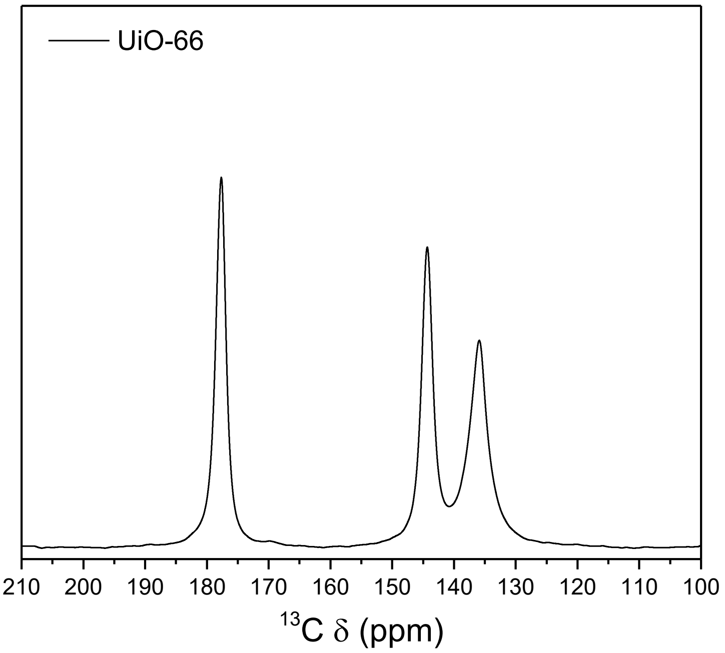

Figure S1. ^{13}C CP-MAS NMR spectrum of the UiO-66.

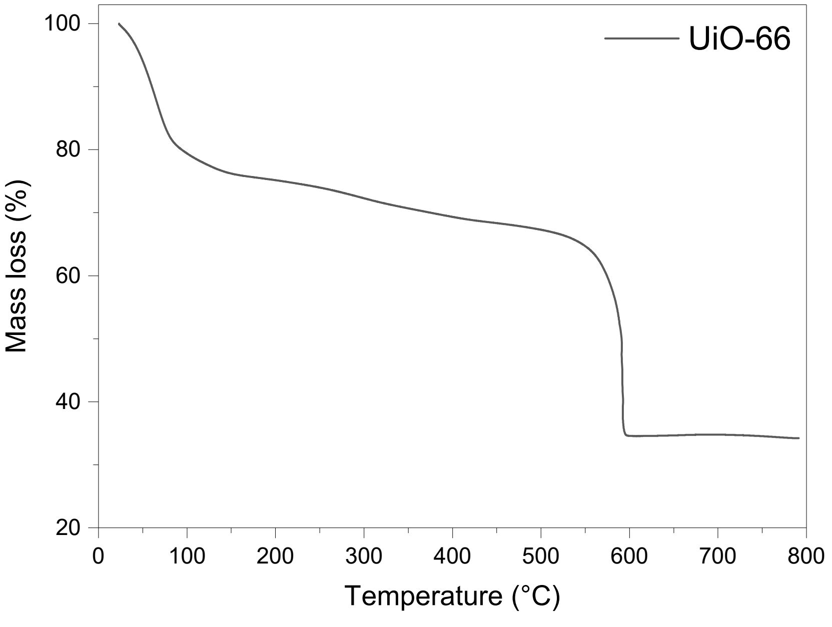

Figure S2. Thermogravimetric curve of the UiO-66.

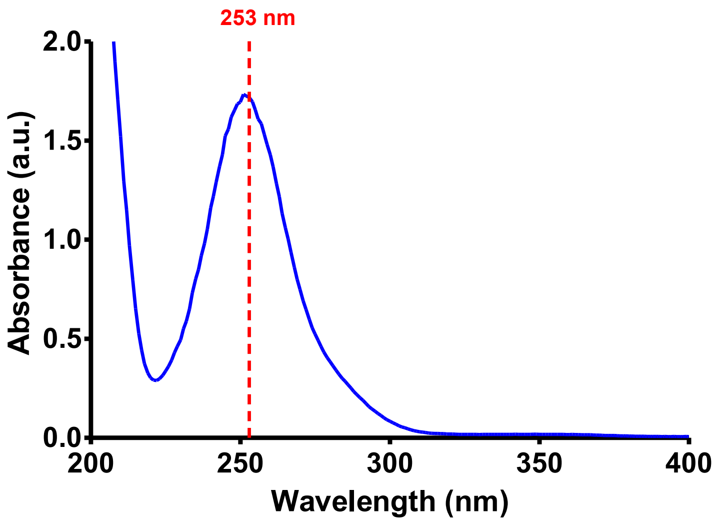

Figure S3. UV-vis spectra of *p*-ASA at pH = 7.0.

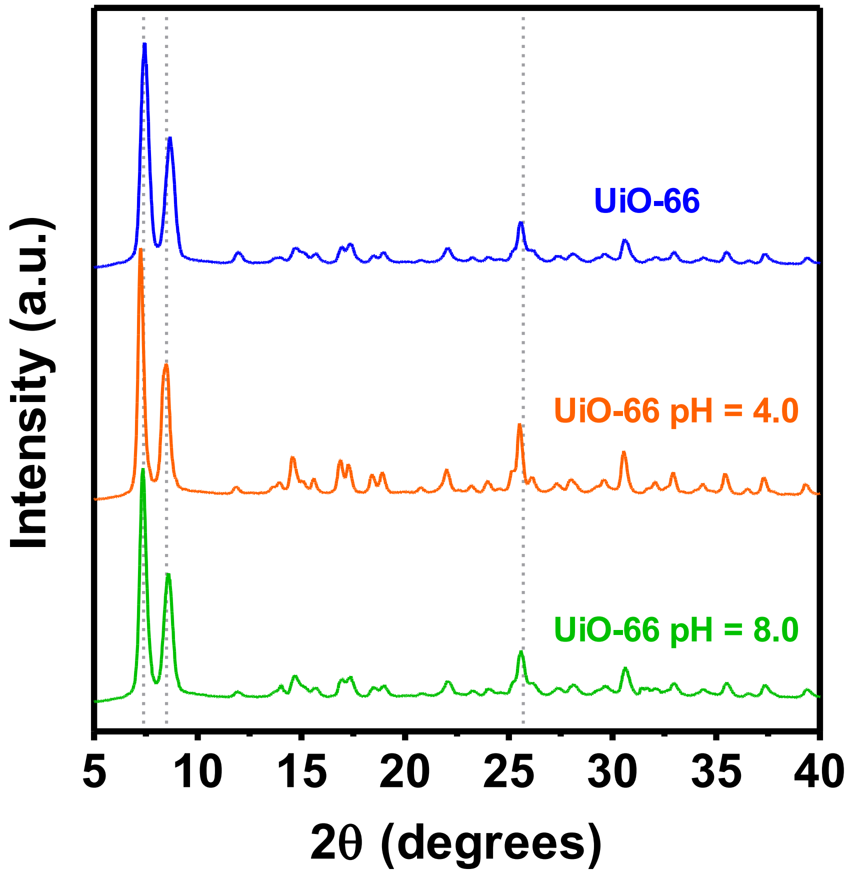

Figure S4. X-ray diffraction patterns of the synthetized UiO-66, UiO-66 at pH 4, and UiO-66 at pH 8.

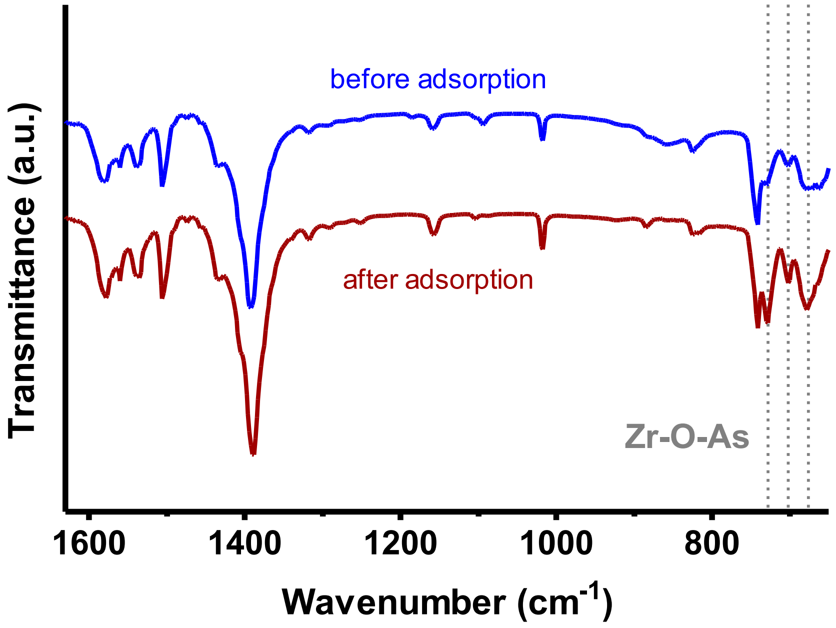

Figure S5. Comparison of FTIR spectra of UiO-66 before and after the adsorption of *p*-ASA.

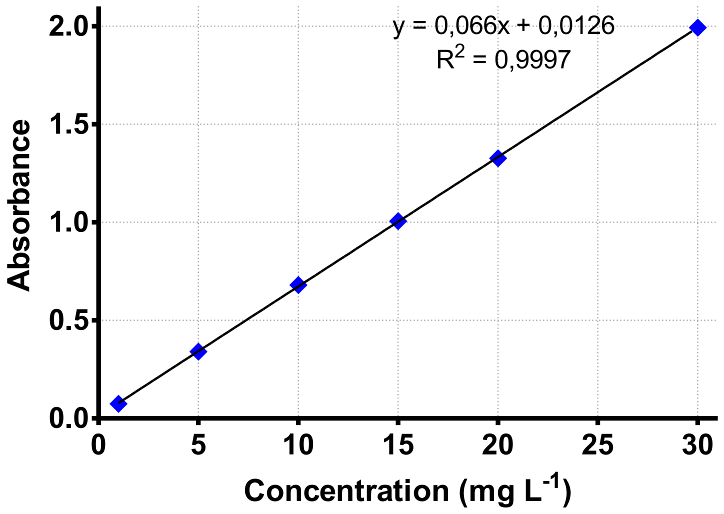

Figure S6. Calibration plot to quantitative measurements of *p*-ASA using UV-Vis spectrometry.
